# Setting the distal and posterior condyle of the femoral component to restore the medial pre-arthritic femoral articular surface results in better outcomes after total knee arthroplasty

**DOI:** 10.1007/s00167-023-07576-9

**Published:** 2023-09-23

**Authors:** Dominik Rak, Thorsten Rügamer, Lukas Klann, Alexander J. Nedopil, Maximilian Rudert

**Affiliations:** Department of Orthopaedic Surgery, König-Ludwig-Haus, University of Würzburg, Würzburg, Germany

**Keywords:** Knee arthroplasty, Clinical outcome, Deviation, Pre-arthritic articular surface

## Abstract

**Purpose:**

The present study of total knee arthroplasty (TKA) describes an intra-operative method that determines the direction and quantifies the magnitude of deviation of the distal and the posterior medial and lateral (DM, PM, DL, and PL) condyle of the femoral component relative to the pre-arthritic femoral articular surface. For each femoral condyle, the deviations were categorized, and an analysis determined which had better or worse Forgotten Joint Score (FJS), Oxford Knee Score (OKS), and WOMAC scores at 1-year follow-up.

**Methods:**

Four academic arthroplasty surgeons supervised a cemented primary CR TKA (Triathlon, Stryker) on 120 consecutive patients. 103 that completed patient-reported outcome measures (PROMs) were analyzed. The surgeon determined the direction and the magnitude of deviation of the condyle of the femoral component by intraoperatively measuring the thickness of the femoral resection, adding compensations of 1 mm for the saw kerf and 2 mm for worn cartilage, and then subtracting the thickness of the femoral component’s condyle. For each femoral condyle, a Kruskal–Wallis test determined the categories of deviation with clinically important and significantly different 1-year PROMs.

**Results:**

A 1 to 2.5 mm and 3 mm or more proximal deviation of the DM condyle of the femoral component worsened the median FJS by 35 and 40 points, OKS by 9 and 14 points, and WOMAC score by 9 and 17 points, respectively, relative to those with a –0.5 to 0.5 mm deviation (*p* < 0.01). A 1 to 2.5 mm and 3 mm or more anterior deviation of the PM condyle of the femoral component worsened the FJS by 34 and 48 points, OKS by 7 and 13 points, and WOMAC scores by 8 and 16 points, respectively (*p* < 0.01). Deviations of the DL and PL condyle of the femoral component did not affect PROMs (*p* ≥ 0.13).

**Conclusions:**

Although many factors can affect PROM, such as patient expectations, the surgeon should understand that setting the DM and the PM condyles of the femoral component within 1 mm of the patient’s pre-arthritic femoral articular surface can potentially result in better FJS, OKS, and WOMAC scores at 1 year.

**Level of evidence:**

II, Prospective cohort study

## Introduction

In total knee arthroplasty (TKA), targets of mechanical alignment (MA), functional alignment (FA), restricted kinematic alignment (rKA), and inverse kinematic alignment (iKA) are restricted to specific limb alignment and component orientation boundaries, which means they can potentially change the femoral and tibial phenotype in the coronal plane [12]. A recent study reported that limiting the change of the femoral phenotype to within one category resulted in a clinically important improvement in patient-reported outcome scores (PROMs) of 41 points for the Forgotten Joint Score (FJS) (100 best, 0 worst), 8 points for the Oxford Knee Score (OKS) (48 best, 0 worst), and 16 points for the Western Ontario and McMaster Universities Osteoarthritis Index (WOMAC) (0 best, 96 worst) relative to those with more than one category change at 1-year [17]. In addition, in the axial plane, each alignment strategy mentioned above can change the rotation of the femoral component relative to the patient’s pre-arthritic posterior joint line or condylar axis, which might potentially affect PROMs.

Intraoperatively quantifying the deviations of the distal medial (DM), posterior medial (PM), distal lateral (DL), and posterior lateral (PL) condyles of the femoral component to within 0.5 mm relative to the pre-arthritic articular surface is a more refined analysis than a postoperative radiographic measurement of a phenotype change and has the advantage of being intraoperatively correctable. For example, the surgeon can compute a value that quantifies the direction and magnitude of deviation of each condyle of the femoral component by intraoperatively measuring the thickness of the corresponding femoral resection, adding compensations of 1 mm for the saw kerf and 2 mm for worn cartilage, and then subtracting the thickness of the femoral component’s condyle. In the coronal and axial planes, a positive/negative value indicates a proximal/distal and anterior/posterior deviation of the condyle of the femoral component relative to the pre-arthritic articular surface, respectively. The computation excludes a compensation for cement because there is no reliable method to measure its thickness in localized regions under each condyle of the femoral component and because it is a systematic error. Finally, a possibility exists that soft tissue releases and the variability in the thickness between the medial and lateral tibial resection could mitigate or accentuate any adverse effects on PROMs induced by a deviation of the DM, PM, DL, or PL condyle of the femoral component from the pre-arthritic articular surface.

The present study asked each of four academic arthroplasty surgeons at a tertiary regional medical center to supervise thirty consecutive TKAs and intraoperatively record the thickness and articular cartilage condition of the distal and posterior femoral resections and the medial and lateral thickness of the tibial resection as an adjunct to their standard technique. The purposes of the study were to determine 1) whether any categories of deviation of DM, PM, DL, and PL condyle of the femoral component relative to the pre-arthritic femoral articular surface had better or worse 1-year FJS, OKS, and WOMAC scores, and 2) whether soft tissue release, the thickness of the medial tibial resection minus the lateral tibial resection, and preoperative patient characteristics of age, body mass index (BMI), knee extension, and knee flexion affect 1-year PROMs.

## Materials and methods

With institutional review board approval (IRB-189/19), the lead author (DR) enlisted four experienced academic TKA specialists to each supervise 30 consecutive cemented primary TKAs for end-stage osteoarthritis using conventional manual instrumentation and a posterior cruciate ligament (PCL) retaining implant design (Triathlon Stryker, Kalamazoo, Michigan, USA). Excluded were patients with avascular necrosis, septic arthritis, prior intra-articular fracture, or a severe pre-operative knee deformity that required revision components to restore stability. In addition, they recorded the patient’s age, pre-operative BMI, knee extension, knee flexion, OKS, and WOMAC. An a priori sample size calculation was based on suppositions that a clinically important deviation of the condyle of the femoral component was ≤ 2 mm or > 2 mm relative to the femoral pre-arthritic articular surface and that there would be an equal number of patients with these deviations. A 2 mm deviation cut-off was chosen because it changes the tibial compartment force by a factor of two [21]. In addition, the parameters used by the calculation were an *α* = 0.05, a power = 0.90, and a 14-point difference of the FJS between the ≤ 2 mm or > 2 mm groups, which is the reported minimal clinical important difference (MCID) with a 20-point standard deviation [1, 2]. Hence, 44 patients were the smallest sample size for each group. An additional 32 patients were added to allow for an uneven proportion of patients between groups, withdrawal, and incomplete follow-up, so 120 patients were enrolled in the study.

**Fig. 1:**
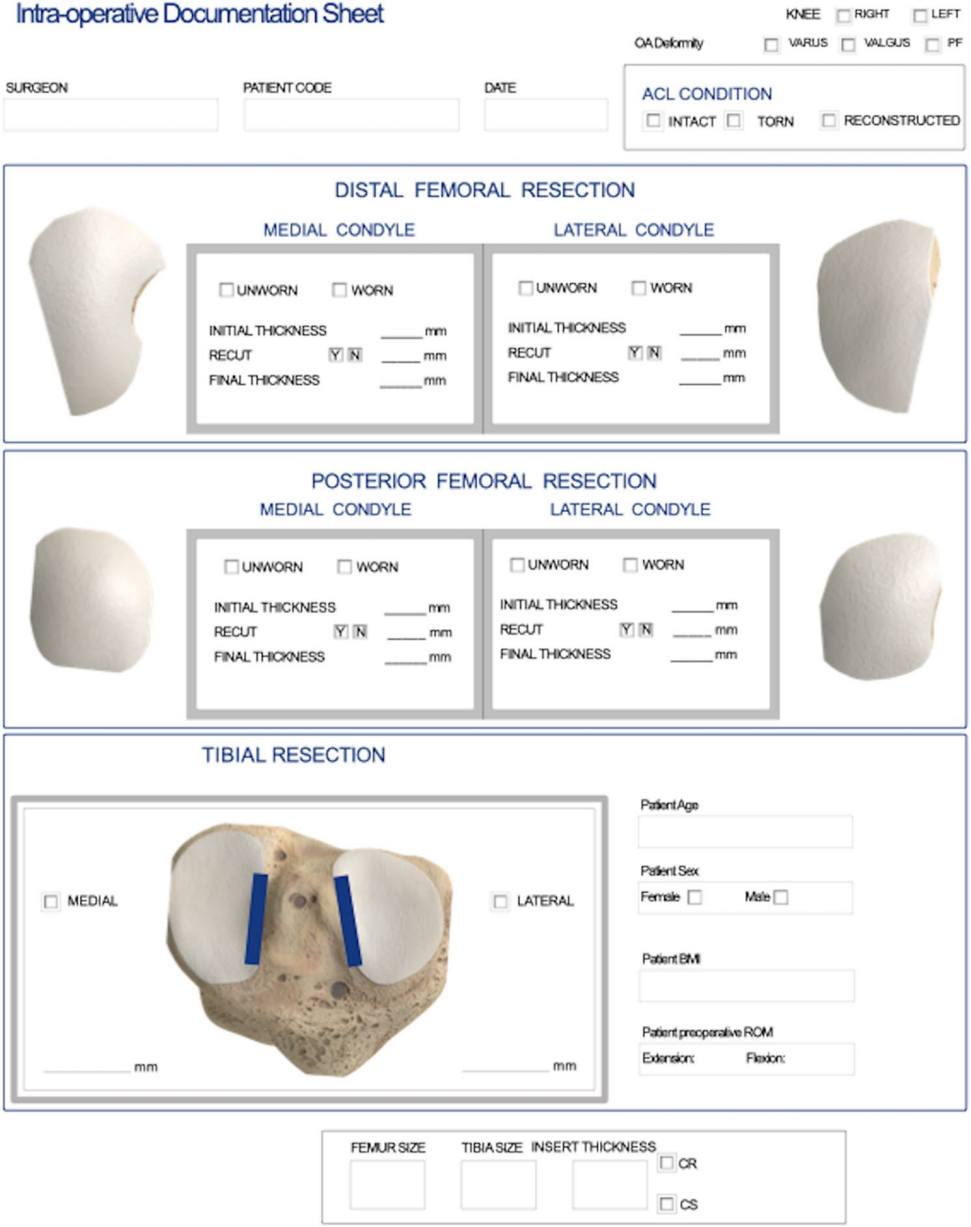
The documentation sheet enabled each surgeon to intraoperatively record the DM, DL, PM, and PL cartilage condition as either worn or unworn and the thickness of each femoral resection as measured to the nearest 0.5 mm with a vernier caliper with flat inside jaws

Each surgeon followed the FDA-approved surgical technique supplied by the manufacturer for the implant design, which was based on mechanical alignment (MA) principles [16]. In addition, they intraoperatively completed a documentation sheet that recorded the knee deformity, femoral cartilage condition, and femoral condyle and tibial bone resection thickness (Fig. 1).

**Fig. 2:**
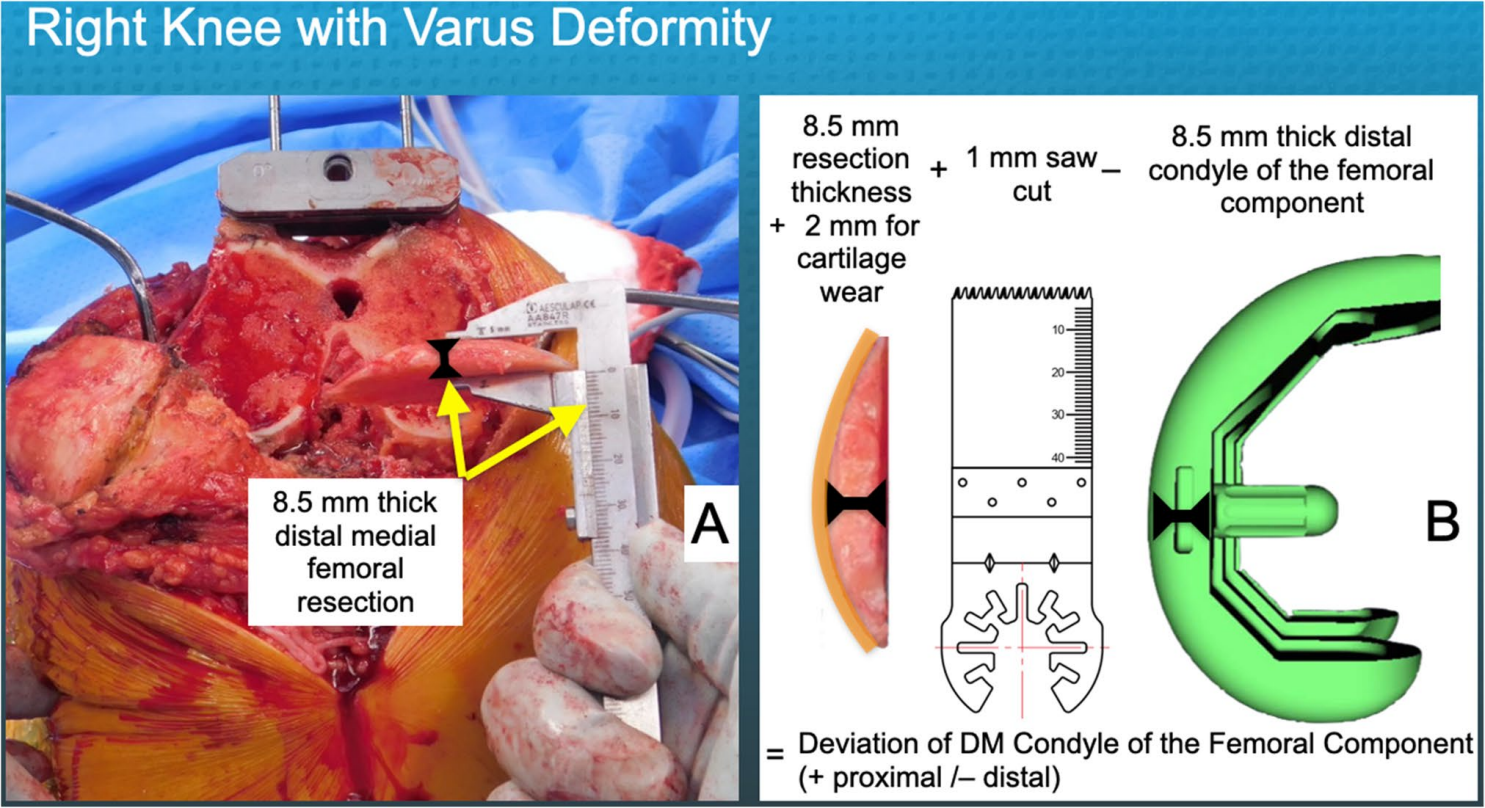
The composite shows a photograph of the thickness measurement of the distal medial resection with a caliper in a right knee with varus deformity and medial cartilage wear (**A**) and a schematic of the computation of the deviation of the distal medial condyle of the femoral component relative to the pre-arthritic articular surface (**B**). The deviation was the caliper thickness of the femoral resection (8.5 mm in this case), plus compensations of 2 mm for cartilage loss (orange curved line) and 1 mm for saw kerf, minus the 8.5 mm thickness of the condyle of the femoral component. Hence, the 8.5 mm resection thickness of the distal medial femur deviated the distal medial condyle of the femoral component 3 mm proximal relative to the patient’s pre-arthritic articular surface

**Fig. 3:**
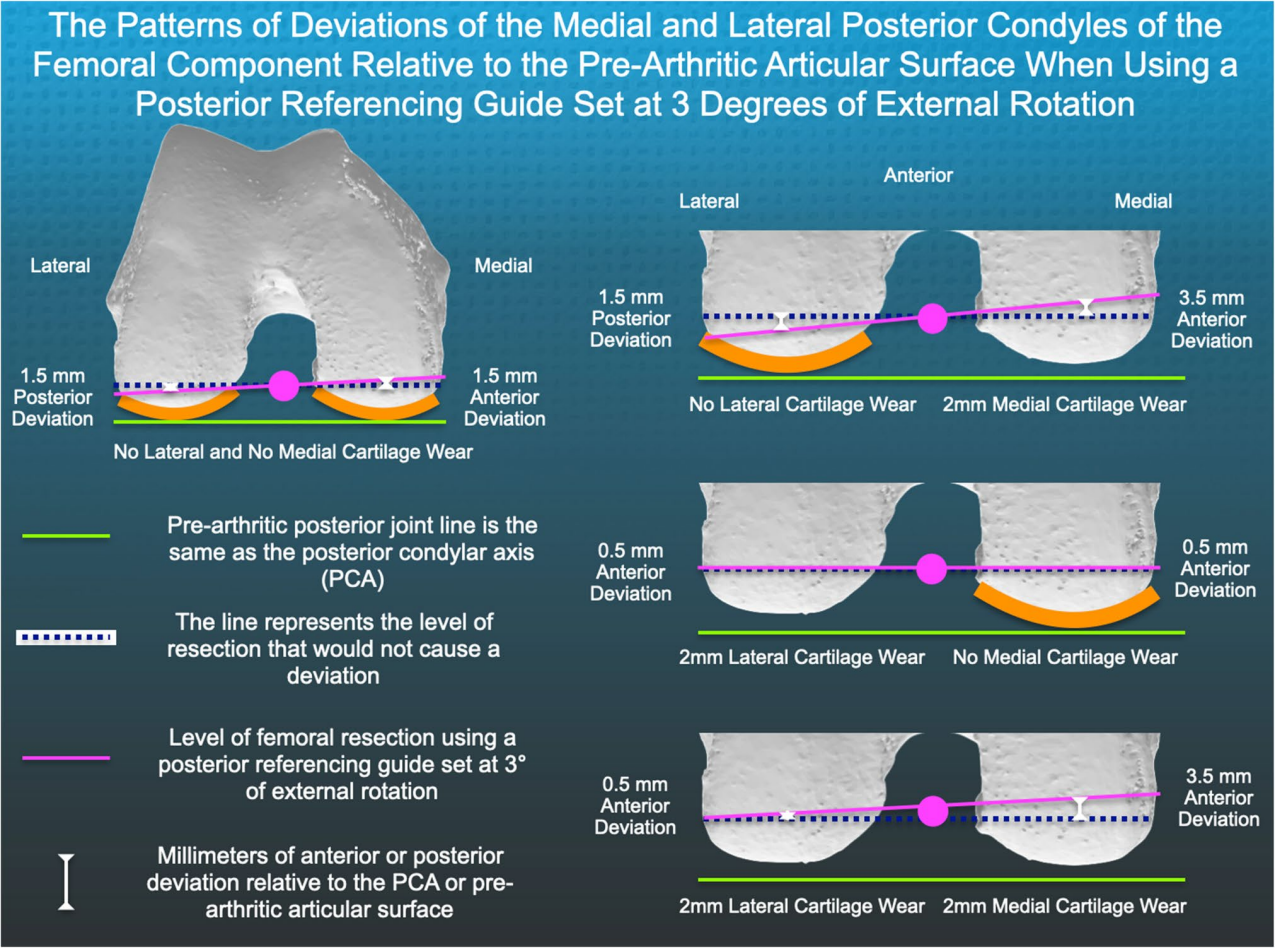
Schematic of an axial view of the distal femur explains the magnitudes and patterns of posterior medial (PM) and posterior lateral (PL) femoral resection patterns reported for the TKAs in the present study using a posterior referencing guide set at 3° external rotation. The presumptions for the analysis are that external rotation occurs about the center of the posterior referencing guide (magenta circle), a 1-degree change in rotation corresponds to a 1 mm change in resection thickness, and the thickness of the posterior cartilage averages 2 mm [14]

The following briefly describes the surgical technique and intra-operative data collection. After exposing the knee, the surgeon classified the primary location of the osteoarthritis as medial (i.e., varus deformity), lateral (i.e., valgus deformity), or patellofemoral. Each surgeon provisionally set the intramedullary distal femoral resection guide at 6° valgus and seated the guide flush with the most distal femoral condyle. The valgus angle was increased to 7° when the projected saw cut was distal to the lateral femoral condyle. After resecting the distal femur, DM and DL articular surface was graded as having intact or missing cartilage, and any partially worn cartilage was removed from the bone. The maximum thickness of the DM and DL resections was measured using a vernier caliper with a resolution of ± 0.5 mm (Fig. 2). No patient had additional bone removed from the distal femur to compensate for a pre-operative flexion contracture. The resection of the posterior femur was performed using the measured resection technique. The posterior referencing guide was set at 3° external rotation relative to and seated flush with PM and PL femoral condyles (Fig. 3). After resecting the posterior femur, the PM and PL articular surface was graded as having intact or missing cartilage, and any partially worn cartilage was removed to the bone. The maximum thickness of the PM and PL resections was measured using a vernier caliper. No patient had additional bone removed from the posterior femur. The proximal tibia was resected perpendicular to the tibial mechanical axis. The thickness of the resection of the medial and lateral tibial compartments was measured at the base of the tibial spines (Fig. 1). The decision to resurface the patella and perform a soft tissue release was left to each surgeon’s discretion. The assessment of soft tissue balance was made qualitatively by clinical laxity testing without using a ligament tensor or measuring tibial compartment forces with a sensor [22]. A review of each patient’s operative note provided the types of soft tissue release.

Weight-bearing long-leg radiographs (LLR) were obtained pre-operatively on all 103 patients. A postoperative weight-bearing LLR was obtained in those patients with full knee extension and who were discharged on a weekday. Those patients discharged during weekends did not have LLR due to a lack of X-ray technicians. Hip–knee–ankle, femoral, and tibial angles were measured and categorized using the functional phenotype classification scheme [10, 12].

The surgeon sent each patient the FJS, OKS, and WOMAC questionnaires one year postoperatively. Those that returned a filled-out questionnaire were included in the study.

### Statistical analysis

The intra-class correlation (ICC) was computed for the four femoral resection measurements using independent measurements made by two observers (i.e., an arthroplasty surgeon and physician assistant) on ten patients (JMP Pro, 17.1.0, http://www.jmp.com, access date July 26, 2023). The ICC values were 0.96/0.98/0.99/1.00 for the DM/DL/PM/PL femoral resections indicating excellent agreement between the two observers.

The Shapiro–Wilk test determined the normality of the dependent variables. Mean ± SD, median, and interquartile range (IQR) described dependent variables with normal and non-normal distributions, respectively. Wilcoxon/Kruskal–Wallis test determined which categories of deviation of the condyle of the femoral component and whether TKAs with or without soft tissue release had significantly better or worse 1-year PROMs. Simple regression determined the significance and the strength of the association, defined as the coefficient of determination (*r*^2^), between thickness of the medial tibial resection minus the lateral tibial resection, preoperative patient characteristics of age, BMI, knee extension, knee flexion, and 1-year PROMs. Significance was set at *p* < 0.05.

## Results

The analysis comprised 103 patients who returned the 1-year PROMs. Preoperative patient characteristics, function scores, and deviations of the condyles of the femoral component were not significantly different between patients with and without 1-year follow-up (*p* = n.s.) (Table 1). There was no difference in the pre-operative and postoperative patient characteristics between surgeons (*p* = n.s.) (Table 2). Hence, the study combined the data from the 103 patients for analysis.

**Table 1:** Preoperative patient characteristics and function scores, and femoral component condyle deviations relative to the pre-arthritic articular surface for the 103 patients with 1-year follow-up and the 17 without

	Patients with 1-year follow-up	Patients without 1-year follow-up	*p*-value
Number of patients (*n*)	103	17	
Patients’ preoperative characteristics and function scores			
Number of patients (*n*)			
Female/Male	48/55	11/6	*p* = n.s.**
Preoperative deformity: varus/valgus/patellofemoral	80/21/2	14/3/0	*p* = n.s.**
Kellgren–Lawrence classification: Grade I/II/III/IV	0/5/29/69	0/0/6/11	*p* = n.s.**
Ahlbäck classification: Grade I/II/III/IV/V	25/22/44/11/1	3/4/8/2/0	*p* = n.s.**
Mean ± Standard deviation (SD)			
Age (years)	67 ± 10	69 ± 11	*p* = n.s.*
BMI^1^	31 ± 7	30 ± 6	*p* = n.s.*
Knee extension (deg)	3 ± 5	3 ± 5	*p* = n.s.*
Knee flexion (deg)	108 ± 11	104 ± 8	*p* = n.s.*
Oxford knee score (48 best, 0 worst)	21 ± 6	19 ± 6	*p* = n.s.*
WOMAC^2^ (0 best, 96 worst)	42 ± 17	46 ± 19	*p* = n.s.*
Mean ± SD of deviation of femoral component condyle relative to pre-arthritic articular surface (+ proximal/anterior, −distal/posterior)			
Distal medial condyle	1.8 ± 1.1	1.4 ± 1.7	*p* = n.s.*
Posterior medial condyle	2.0 ± 1.4	1.8 ± 1.4	*p* = n.s.*
Distal lateral condyle	−0.3 ± 1.7	−0.9 ± 1.5	*p* = n.s.*
Posterior lateral condyle	−0.1 ± 1.7	0.8 ± 2.2	*p* = n.s.*

^1^Body Mass Index

^2^Western Ontario and McMaster Universities Arthritis Index

^*^T-Test determined differences between patients with and without 1-year follow-up

^**^Fisher’s exact test determined differences between patients with and without 1-year follow-up

**Table 2:** Pre- and postoperative patient characteristics and function scores were comparable for the patients treated by each surgeon

	Surgeon 1	Surgeon 2	Surgeon 3	Surgeon 4	*p*-value
Number of patients (*n*) with 1-year follow-up	27	27	28	21	
Years of practice	30	22	17	14	
Patients’ preoperative characteristics and function scores					
Number of patients (*n*)					
Female/Male	14/13	15/12	15/12	8/13	*p* = n.s.**
Preoperative deformity: varus/valgus/patellofemoral	21/6/0	18/8/1	25/2/1	16/5/0	*p* = n.s.**
Kellgren–Lawrence classification: Grade I/II/III/IV	0/1/5/21	0/1/7/19	0/3/9/16	0/0/8/13	*p* = n.s.**
Ahlbäck classification: Grade I/II/III/IV/V	5/4/11/6/1	5/7/13/2/0	9/9/9/1/0	6/2/11/2/0	*p* = n.s.**
Mean ± Standard deviation					
Age (years)	70 ± 8	64 ± 9	66 ± 9	67 ± 11	*p* = n.s.*
BMI^1^	31 ± 7	32 ± 8	30 ± 6	30 ± 6	*p* = n.s.*
Knee extension (deg)	4 ± 5	3 ± 6	3 ± 5	3 ± 4	*p* = n.s.*
Knee flexion (deg)	109 ± 10	107 ± 12	109 ± 10	109 ± 10	*p* = n.s.*
Oxford knee score (48 best, 0 worst)	22 ± 6	20 ± 6	22 ± 5	19 ± 5	*p* = n.s.*
WOMAC^2^ (0 best, 96 worst)	43 ± 16	48 ± 16	43 ± 19	49 ± 15	*p* = n.s.*
Median and [IQR]^3^ of postoperative function scores					
Forgotten joint score (100 best, 0 worst)	43 [27–77]	50 [29–73]	36 [23–70]	58 [27–74]	*p* = n.s.***
Oxford knee score (48 best, 0 worst)	36 [31–41]	40 [33–43]	34 [30–41]	42 [30–45]	*p* = n.s.***
WOMAC^2^ (0 best, 96 worst)	16 [6–30]	11 [4–18]	14 [8–29]	9 [6–22]	*p* = n.s.***

^1^Body Mass Index

^2^Western Ontario and McMaster Universities Arthritis Index

^3^Interquartile Range

^*^ANOVA determined differences between surgeons

^**^Pearson’s chi-Square Test determined differences between surgeons

^***^Kruskal–Wallis Test determined differences between surgeons

**Fig. 4:**
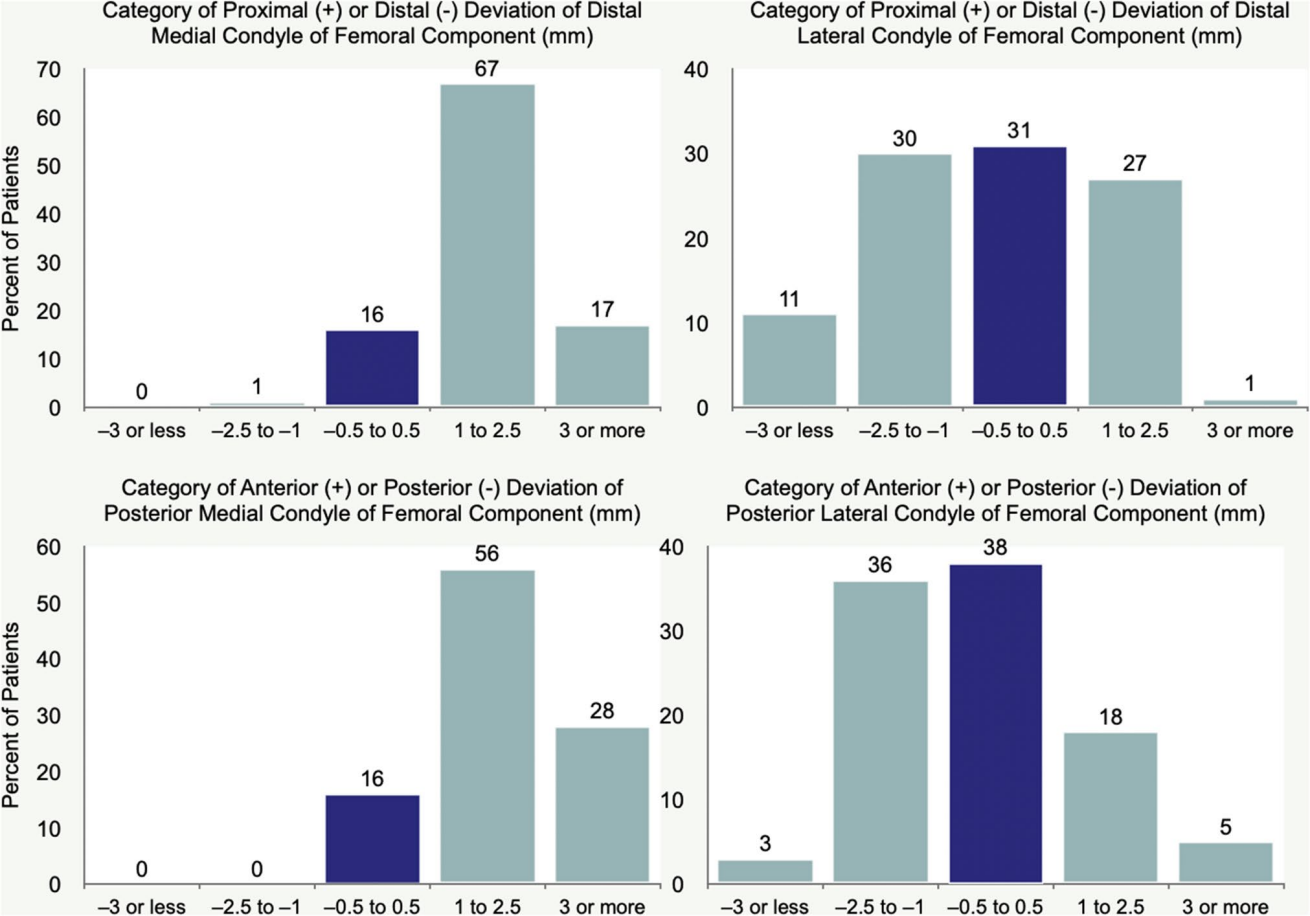
The distributions show the proportion of patients assigned to one of five categories based on the deviation of the DM, DL, PM, and PL condyle of the femoral component relative to the pre-arthritic articular surface. For example, the blue column indicates the proportion of patients with a minor deviation of −0.5 to 0.5 mm

**Fig. 5:**
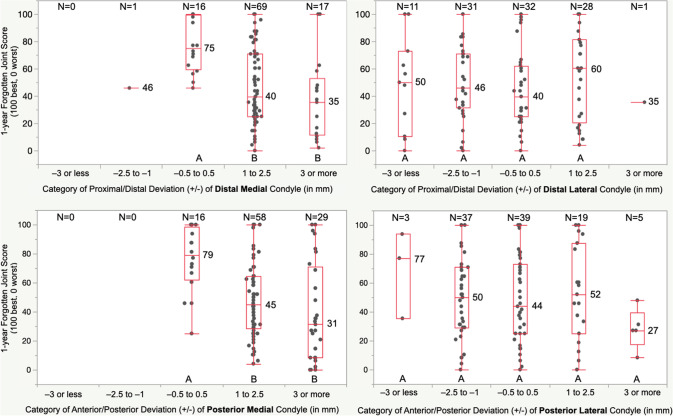
The boxplot of the deviation of the DM, DL, PM, and PL condyle of the femoral component shows the median FJS (number by transverse line in each box) for each category of deviation. Deviations denoted by different capital letters were significantly different. For example, those patients with a proximal and anterior deviation of the DM and PM condyle of 1 to 2.5 mm and 3 mm or more had a significantly lower median FJS at least two times the 14-point MCID relative to those with a restored pre-arthritic articular surface (−0.5. to 0.5 mm) (*p* < 0.0011)

**Fig. 6:**
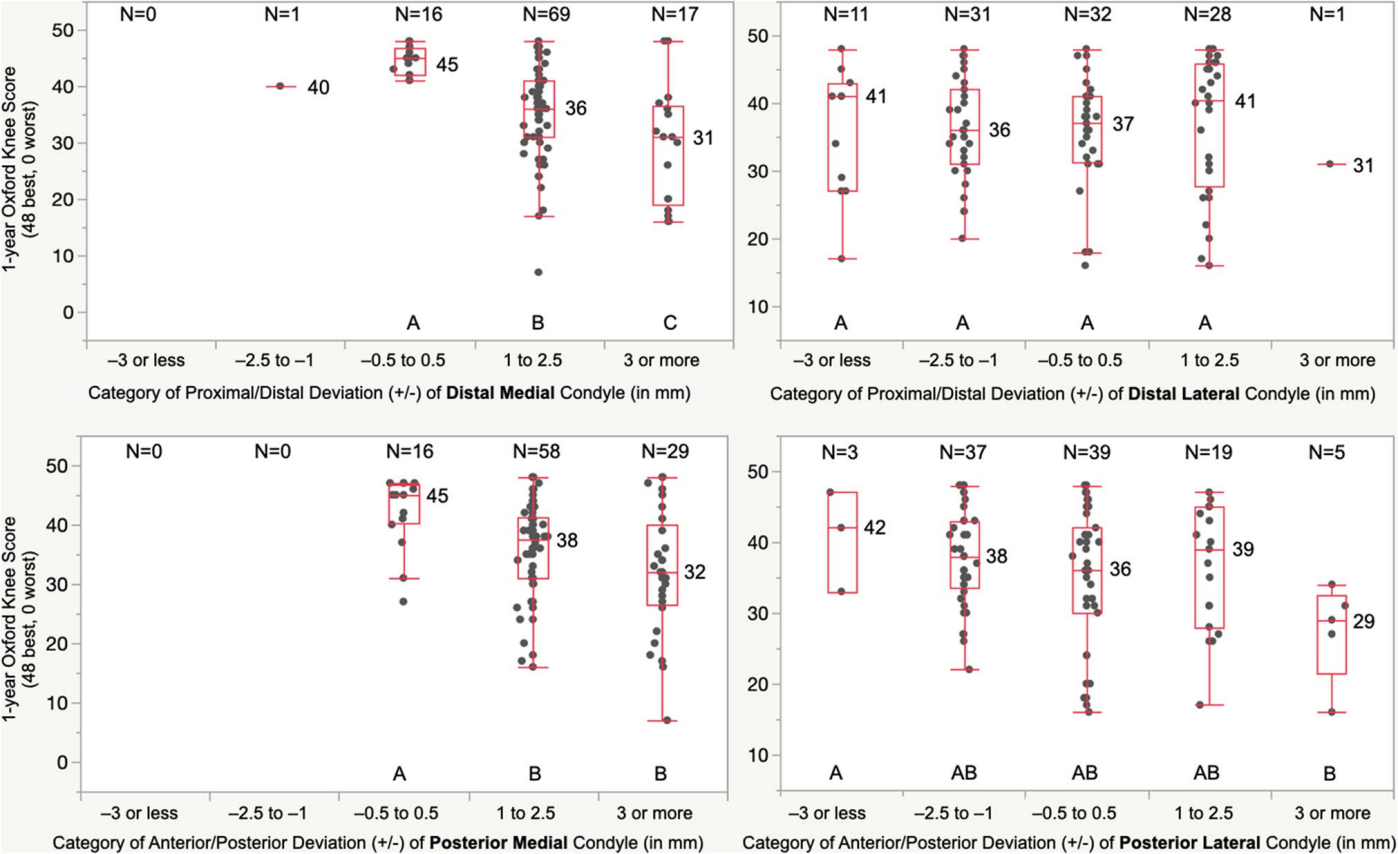
The boxplot of the deviation of the DM, DL, PM, and PL condyle of the femoral component, shows the median OKS for each category of deviation. For example, those patients with a proximal and anterior deviation of the DM and PM condyle of 1 to 2.5 mm and 3 mm or more had a significantly lower OKS by at least 1 to 2 times the 5-point MCID relative to those with a restored pre-arthritic articular surface (−0.5. to 0.5 mm) (*p* < 0.0027)

**Fig. 7:**
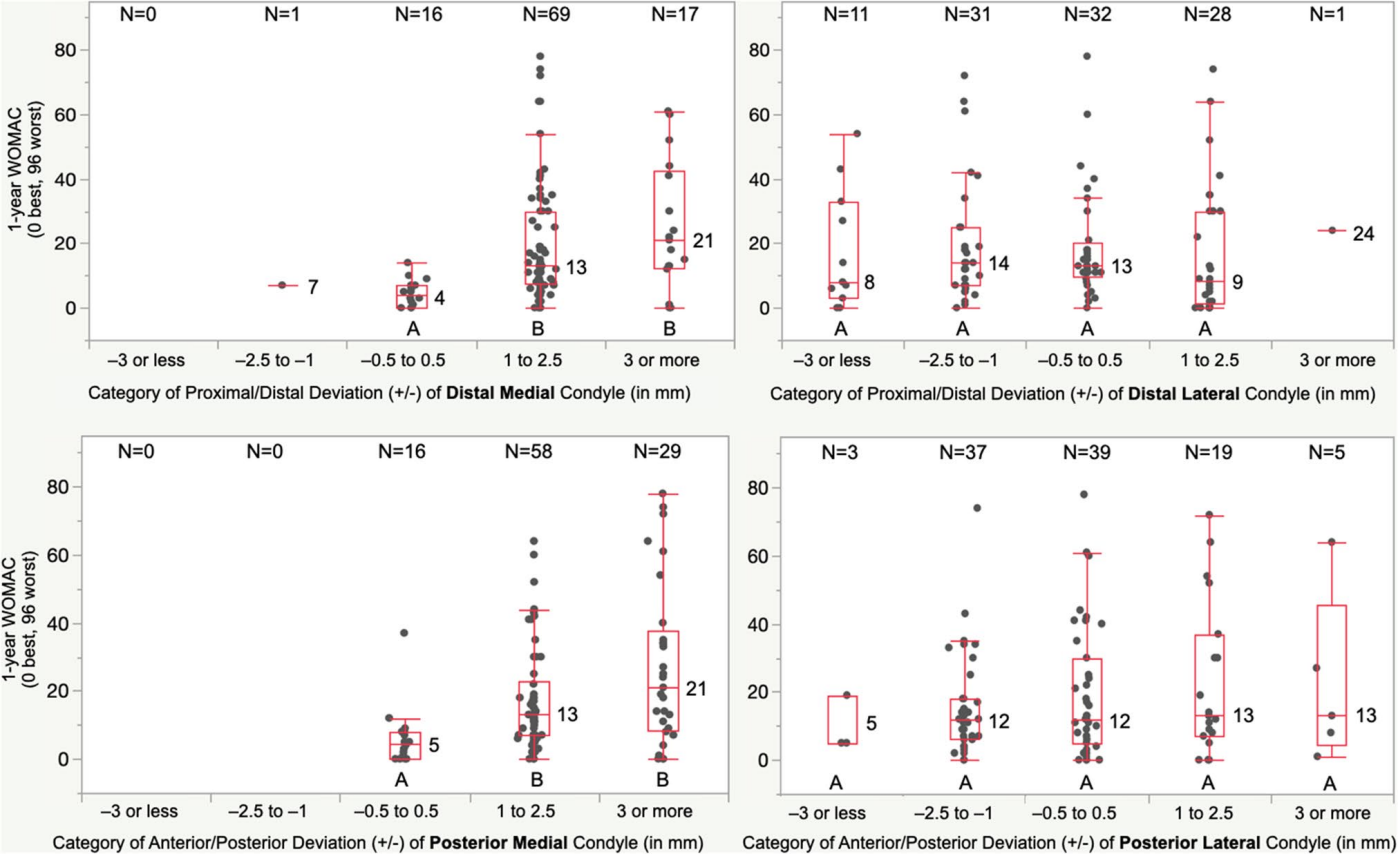
The boxplot of the deviation of the DM, DL, PM, and PL condyle of the femoral component, shows the median WOMAC score for each category of deviation. For example, those patients with a proximal and anterior deviation of DM and PM condyle of 1 to 2.5 mm and 3 mm or more had a significantly higher WOMAC score by 8 to 17 points (i.e., 10-point MCID) relative to those with a restored pre-arthritic articular surface (*p* < 0.0008)

The proportion of patients within each category of deviation of the condyle of the femoral component was similar for DM and PM femoral articular surfaces and different from the DL and PL, which were similar (Fig. 4). A 1 to 2.5 mm and 3 mm or more proximal deviation of the DM condyle of the femoral component worsened the median FJS by 35 and 40 points, OKS by 9 and 14 points, and WOMAC score by 9 and 17 points, respectively, relative to those with a −0.5 to 0.5 mm deviation (*p* < 0.01). A 1 to 2.5 mm and 3 mm or more anterior deviation of the PM condyle of the femoral component worsened the FJS by 34 and 48 points, OKS by 7 and 13 points, and WOMAC scores by 8 and 16 points, respectively (*p* < 0.01). Deviations of the DL and PL condyle of the femoral component did not affect PROMs (*p* > 0.13). (Figs. 5, 6, and 7). Types and distribution of soft tissue releases are provided in Table 3. The median 1-year FJS, OKS, and WOMAC of the 34 patients with a soft tissue release of 45, 36, and 14 points were not significantly different from the scores of 48, 39, and 12 points of the 69 patients without soft tissue release (*p* = n.s.). The thickness of the medial tibial resection minus the lateral tibial resection, preoperative patient characteristics of age, BMI, knee extension, and knee flexion poorly predicted the 1-year PROMs (Table 4).

**Table 3:** Types of soft tissue releases performed and distribution of patients according to the type of soft tissue release

Type of soft tissue release	None	Medical collateral ligament (MCL)	Partial posterior cruciate ligament (PCL)	Popliteus tendon	Iliotibial band + Patellofemoral ligament	MCL + partial PCL	Popliteus tendon + Patellofemoral ligament
Number of patients (N)	69	20	9	2	1	1	1
% of included patients	67%	19%	9%	2%	1%	1%	1%

**Table 4:** Comparison of PROMs in patients without and with soft tissue release during TKA and associations of thickness of the medial tibial resection minus the lateral tibial resection, preoperative patient characteristics of age, BMI, knee extension, and knee flexion with PROMs

	Forgotten joint score	Oxford knee score	WOMAC^1^
Median and [IQR]^2^ of postoperative function scores			
No soft tissue release (*n* = 69)	48 [25;71]	39 [31;44]	12 [5;23]
Soft tissue release performed (*n* = 34)	45 [27;74]	36 [29;40]	14 [7;35]
Significance of difference	*p* = n.s.*	*p* = n.s.*	*p* = n.s.*
Strength of association (*r*^2^); Significance (*p*-value)			
Thickness of the medial tibial resection minus the lateral tibial resection	*r*^2^ < 0.01; * p* = n.s.**	*r*^2^ < 0.01; * p* = n.s.**	*r*^2^ < 0.01; * p* = n.s.**
Age	*r*^2^ = 0.02; * p* = n.s.**	*r*^2^ = 0.01; * p* = n.s.**	*r*^2^ < 0.01; * p* = n.s.**
BMI^1^	*r*^2^ = 0.02; * p* = n.s.**	*r*^2^ = 0.02; * p* = n.s.**	*r*^2^ < 0.01; * p* = n.s.**
Preoperative knee extension	*r*^2^ = 0.03; * p* = n.s.**	*r*^2^ = 0.02; * p* = n.s.**	*r*^2^ < 0.01; * p* = n.s.**
Preoperative knee flexion	*r*^2^ < 0.01; * p* = n.s.**	*r*^2^ < 0.01; * p* = n.s.**	*r*^2^ < 0.01; * p* = n.s.**

^1^Western Ontario and McMaster Universities Arthritis Index

^2^Interquartile Range

^*^Kruskal–Wallis Test determined differences between TKAs with and without soft tissue release

^**^F-test determined significance of association

Table 5 shows the distribution of 103 patients according to preoperative limb, femoral, and tibial phenotypes relative to reported phenotype distributions in the osteoarthritic population and the distribution of the TKA phenotypes for the 59 patients with postoperative LLR [10].

**Table 5:** Distribution of preoperative and postoperative TKA functional phenotypes in the present study relative to phenotype distributions reported for the osteoarthritic knee

Study (number of patients)	Functional phenotypes						
	VAR ≥ 9°	VAR6°	VAR3°	NEU0°	VAL3°	VAL6°	VAL ≥ 9°
Preoperative osteoarthritic limb phenotypes (HKA) (%)							
Present study (*n* = 103)	14	33	25	6	7	4	11
Hess et al. [10] (*n* = 2692)	22	25	21	13	9	6	4
Preoperative osteoarthritic femoral phenotype (FMA) (%)							
Present study (*n* = 103)		10	22	46	23		
Hess et al. [10] (*n* = 2692)	1	4	28	43	20	4	
Preoperative osteoarthritic tibial phenotype (TMA) (%)							
Present study (*n* = 103)		3	20	39	26	13	
Hess et al. [10] (*n* = 2692)	2	8	29	41	17	3	
Postoperative TKA limb phenotype (HKA) (%)							
Present study (*n* = 59)		3	22	54	19	2	
Postoperative TKA femoral phenotype (FMA) (%)							
Present study (*n* = 59)		22	69	7	2		
Postoperative TKA tibial phenotype (TMA) (%)							
Present study (*n* = 59)			2	22	54	22	

## Discussion

The present study has two significant findings: First, patients with less than a 1 mm deviation of the DM or PM condyle of the femoral component relative to the pre-arthritic femoral articular surface had better one-year FJS, OKS, and WOMAC than those with a 1 mm or more proximal or anterior deviation by a margin of 1 to 2 times greater than the MCID (Figs. 5, 6, and 7). Second, a soft tissue release, the thickness of the medial tibial resection minus the lateral tibial resection, patient preoperative characteristics of age, BMI, knee extension, and knee flexion did not adversely affect the PROMs.

There are several explanations for the proximal deviation of the DM condyle of the femoral component relative to the pre-arthritic articular surface. One explanation is that most patients have a valgus phenotype, and they undergo a varus change relative to the pre-arthritic distal femoral joint line when the resection of the distal femur is 90° relative to the femoral mechanical axis [11, 19, 20]. Another explanation for the proximal deviation of the DM condyle of the femoral component is the seating of the distal femoral resection guide on the worn surface of the medial femoral condyle with missing cartilage.

One explanation for the anterior deviation of the PM condyle of the femoral component is that the posterior referencing guide was set at 3° external rotation. However, the deviation of the PM relative to the patient's pre-arthritic articular surface depends on many additional parameters, such as whether the rotation occurs about the center of the guide, as in the present study, and whether the PM and PL cartilage is intact, which averages 2 mm in thickness [4, 14] (Fig. 3). Because there are so many possibilities of anterior and posterior deviations of PM and PL condyles of the femoral components that are dependent on alignment targets, such as the transepicondylar axis and anteroposterior axis of the trochlear groove, the findings that anterior deviation of the PM condyle of the femoral component resulted in worse PROMs in the present might not be generalizable [3, 8, 9, 15]. However, because of the clinically important lowering of the PROMs caused by a 1 mm or more anterior deviation of the PM condyle of the femoral component, surgeons should consider studying the consequence of this surgical variable, which is just as easily intraoperatively determined, as shown in the present study, when performing MA, FA, rKA, and iKA.

Soft tissue release did not affect the PROMs, indicating that restoring the patient’s medial pre-arthritic articular surface is a more important factor for improving PROMs. In addition, setting the femoral component so it does not resurface the patient’s femur might be the reason for the soft tissue release as this creates a wide range of collateral ligament imbalances [3, 8, 9, 15]. Finally, it is unlikely that the soft tissue release’s lack of effect on PROMs was due to the study being underpowered as the median difference of 3 points in the FJS, 3 points in the OKS, and 2 points in the WOMAC score between the 34 and 69 TKAs with and without a release was each small enough to be clinically unimportant and the unimportant median differences are unlikely to change with a larger sample size.

Finally, with the number of TKA’s available for analysis, other variables such as the difference in the thickness of the medial tibial resection minus the lateral tibial resection, patient preoperative characteristics of age, BMI, knee flexion, and knee extension were not associated with the PROMs, which suggests that the deviation of the DM and PM condyle of the femoral component is a primary determinant of these scores (Table 4). For example, the thickness of the medial tibial resection minus the lateral tibial resection did not compensate for deviations in femoral component placement relative to the pre-arthritic femur, which is consistent with a phenotype analysis that showed changes in tibial phenotype relative to the pre-operative phenotype did not affect the PROMs, whereas changes in femoral phenotype did [17].

The present study confirms the results from virtual planning studies, which demonstrated that MA TKA changes the pre-arthritic distal femoral articular surface ≥ 3 mm in more than 75% of patients and the pre-arthritic posterior femoral articular surface in more than 95% of patients [11, 18, 19]. Alternative alignment strategies like unrestricted KA, rKA, iKA, and FA have emerged to reduce the frequency and magnitude of joint line changes [13, 19, 20]. Except unrestricted KA, these alternative alignment strategies preserve the pre-arthritic femoral joint line orientation only within a safe zone defined by limb alignment, the femoral mechanical angle, and flexion space balance, which still changes the pre-arthritic femoral distal or posterior articular surface in 40% [18–20]. Hence, the best postoperative FJS, OKS, and WOMAC scores occur by minimizing the femoral component’s deviation from the pre-arthritic articular surface to less than 1 mm rather than dogmatically adhering to an alignment strategy that changes the medial femoral pre-arthritic articular surface [5, 6, 17, 19, 22].

The present study has several limitations. First, the present study assessed only one FDA-approved surgical technique provided by the manufacturer for the CR implant, and other alignment versions exist. For example, in the coronal plane, some surgeons prefer to customize the level and the angle of the distal femoral cut based on the arthritic joint line instead of setting the distal resection 8 mm proximal to the most distal femoral condyle with a 6° valgus setting of the distal femoral cutting guide relative to the intramedullary canal, as was done in the present study, whereas in the axial plane, some prefer to set the posterior joint line perpendicular to the, parallel to the transepicondylar axis, anteroposterior axis of the trochlear groove, or parallel to the tibial resection in 90° of flexion with the use of gap-balancing instead of externally rotated 3° relative to the posterior condylar line as in the present study. A second limitation is that bone wear at 0 and 90°, where the guides reference the femur, would have underestimated the proximal and anterior deviation of the DM and PM condyle of the femoral component relative to the patient’s pre-arthritic articular surface. However, an analysis of 208 osteoarthritic knees (154 varus/ 54 valgus) showed mean bone wear at 0° and 90° was 0.0 ± 0.2 mm [14]. Although femoral bone wear can occur in the weight-bearing arc of 5° to 50° during gait [7], the increasing risk of a flexion contracture with increasing Kellgren–Lawrence severity diminishes the risk of femoro-tibial contact and bone wear at the 0° flexion location, which explains why a compensation for bone wear was unnecessary [23].

## Conclusion

Although many factors can affect PROM, such as patient expectations, the surgeon should understand that setting DM and PM condyles of the femoral component within 1 mm of the patient’s pre-arthritic femoral articular surface can potentially result in better FJS, OKS, and WOMAC scores at 1 year.

## Data Availability

The data that support the findings of this study are not openly available due to reasons of sensitivity and are available from the corresponding author upon reasonable request.
